# Myofibroblastic Proliferation as a Secondary Malignancy From Radioactive Iodine Treatment for Thyroid Carcinoma: A Case Report

**DOI:** 10.7759/cureus.90257

**Published:** 2025-08-16

**Authors:** Adeel Riaz, Malay Rao, Mariam Arif, Abu Hurrairah, Varisha A Hussain

**Affiliations:** 1 General Surgery, The Brooklyn Hospital Center, Brooklyn, USA; 2 Radiation Oncology, Rutgers Robert Wood Johnson Medical School, New Brunswick, USA; 3 Radiation Oncology, Cooperman Barnabas Medical Center, Livingston, USA; 4 Internal Medicine, Lincoln Medical and Mental Health Center, Bronx, USA; 5 Radiology, Aziz Fatima Medical and Dental College, Faisalabad, PAK; 6 Medicine, Queens College, City University of New York, Flushing, USA

**Keywords:** myofibroblastic proliferation, radiation risks, radioactive iodine, secondary malignancy, thyroid carcinoma

## Abstract

Thyroid carcinoma is an important health issue having significant morbidity and mortality. Management includes thyroidectomy followed by radioactive iodine ablation to kill any remaining tumor cells. However, the use of radioactive iodine ablation (RAI) is not without risks; one such risk is the development of secondary cancers, especially in patients undergoing radioactive iodine ablation at a younger age. We present a case of thyroid carcinoma where the patient was treated with radioactive iodine ablation over 20 years ago. The patient was recently found to have a neck mass, which was investigated with imaging and biopsied. Pathological findings were consistent with myofibroblastic proliferation, which is a benign abnormal growth of myofibroblasts but has small potential for malignant transformation. Management generally involves surgical excision, and other treatments like steroids, non-steroidal anti-inflammatory drugs, and radiation therapy can be used in select cases

## Introduction

Thyroid cancer is the second most common cancer in the young population under 45 years of age, with incidence increasing over the last several decades at a rate of 3-4% per year. This is largely owing to an increase in the diagnosis of low-risk, smaller tumors [1]. Papillary and follicular thyroid carcinomas are well-differentiated thyroid cancers, constituting more than 90% of thyroid cancers in the United States, with women having three times more likelihood of developing them compared with men. The management of well-differentiated thyroid cancers involves definitive thyroidectomy, followed by radioactive iodine ablation (RAI) of the residual gross or microscopic tumor cells [2].

Although RAI increases the overall survival and disease-free survival (DFS), its role in low- and intermediate-risk thyroid cancers is controversial because of the high five-year DFS of over 97% even without the use of RAI [1,3]. Moreover, the use of RAI is not risk-free. Studies have shown that RAI is associated with increased risk of secondary hematologic and solid tumors. Although rare, these secondary malignancies can result in significant morbidity and mortality. We hereby present a case of a secondary tumor that developed in a patient who underwent RAI ablation ~20 years ago for treatment of thyroid carcinoma. The patient carried a history of thyroid cancer, although records were not available to verify the histologic subtype.

## Case presentation

**Figure 1:**
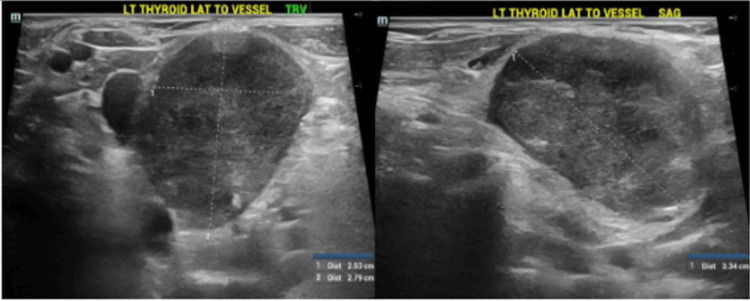
Ultrasonogram of the neck showing a 3.3 x 2.5 x 2.8 cm large solid mass besides the left thyroid lobe lateral to the internal jugular vein

A 79-year-old female with a history of hypertension, mitral valve prolapse, and thyroid cancer (although records were not available to verify the histologic type), treated with RAI treatment (no external beam radiation) more than 20 years ago, which resulted in acquired hypothyroidism. She presented to the endocrine clinic for a routine follow-up and was found to have a left-sided neck mass on physical exam. The mass was soft, mobile, and non-tender, with no signs of acute inflammation. The patient did not notice the mass until found on exam. She did not report pain, dysphagia, odynophagia, hoarseness, or weight loss. Due to her previous history of thyroid cancer, radiation exposure in the form of RAI, and high clinical suspicion, an ultrasonogram of the thyroid was done, which showed a large solid mass lateral to the thyroid and the internal jugular vein measuring (3.3 x 2.5 x 2.8 cm) in size and with increased vascularity (Figure 1).

**Figure 2:**
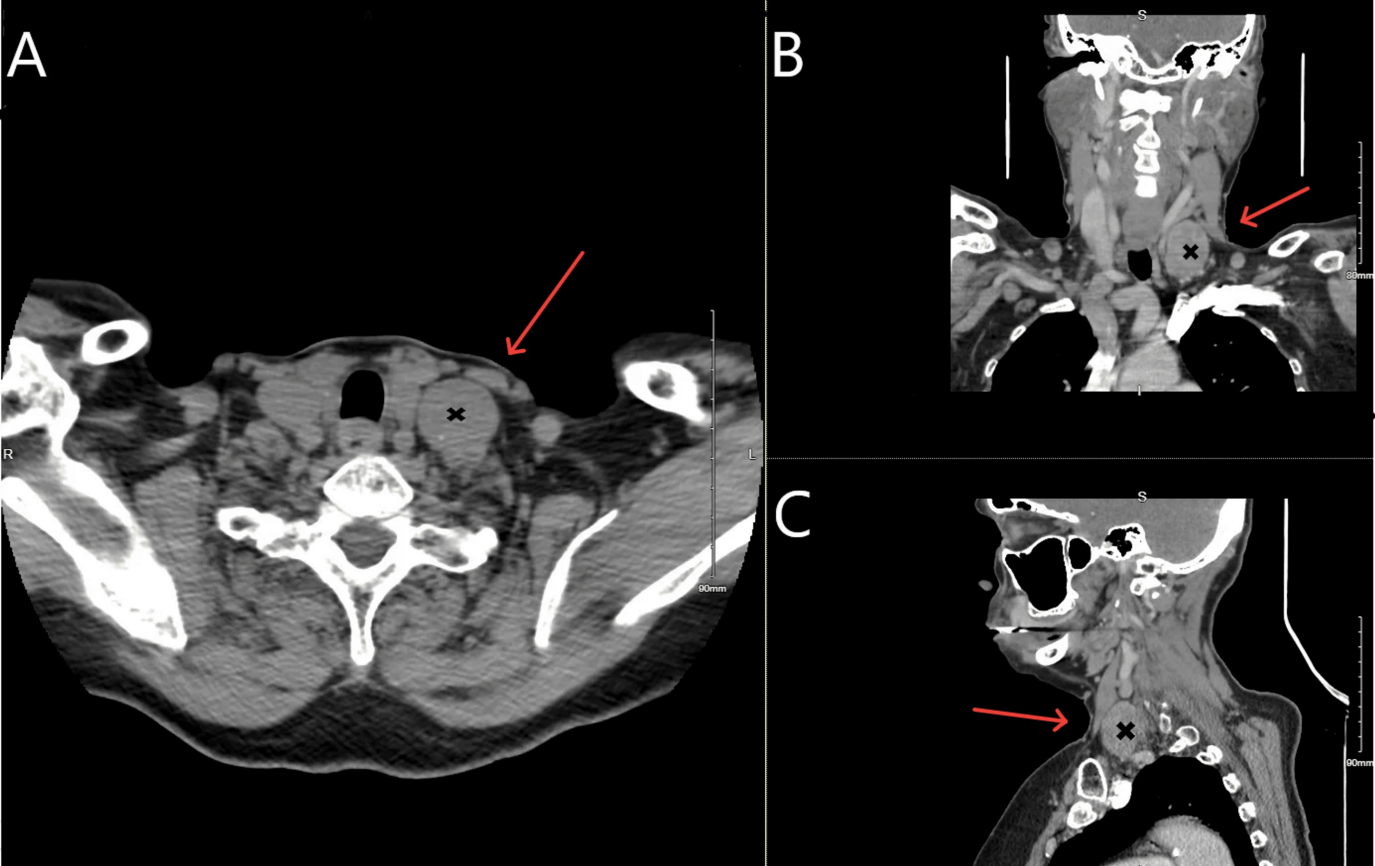
A CT scan of soft tissue neck showing a left neck mass labeled 'X', measuring 4.1 x 2.6 x 3.0 cm in size (red arrows pointing the mass) Panel A showing the axial section, Panel B showing the coronal section, and Panel C showing the sagittal section of the CT soft tissue neck.

For better soft tissue delineation and staging purposes, computed tomography (CT) soft tissue neck and chest were performed that showed a heterogeneously enhancing sharply demarcated ovoid mass measuring 4.1 x 2.6 x 3.0 cm (Figure 2) within the deep left neck adjacent to left thyroid lobe, lateral to the left carotid sheath, at the level of the common carotid artery. Shotty small nodes were noted throughout the neck, but no obvious metastatic disease was noted.

**Figure 3:**
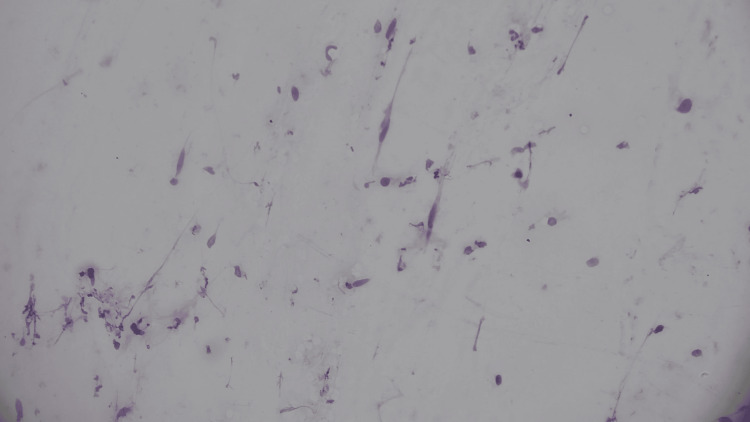
Bland spindle to ovoid cells with scant cytoplasm dispersed as clusters and single cells (touch preparation)

**Figure 4:**
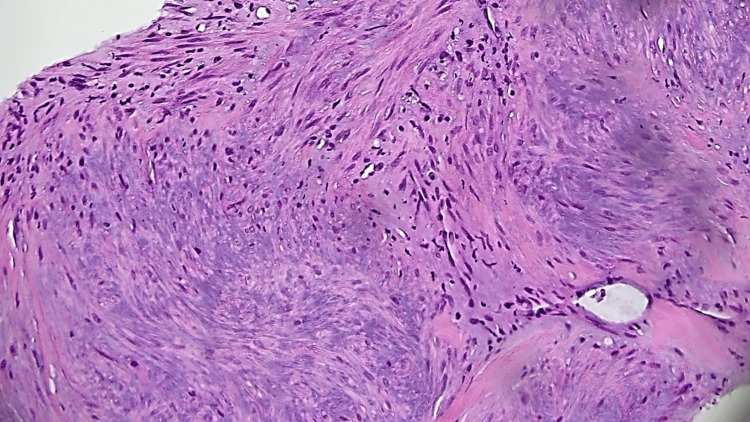
Bland, uniform, short to elongated spindle cells arranged as short haphazard intersecting fascicles admixed with bands of hyalinized, brightly eosinophilic collagen​ (no nuclear atypia, mitoses absent) Hematoxylin and eosin (H&E) 20x

**Figure 5:**
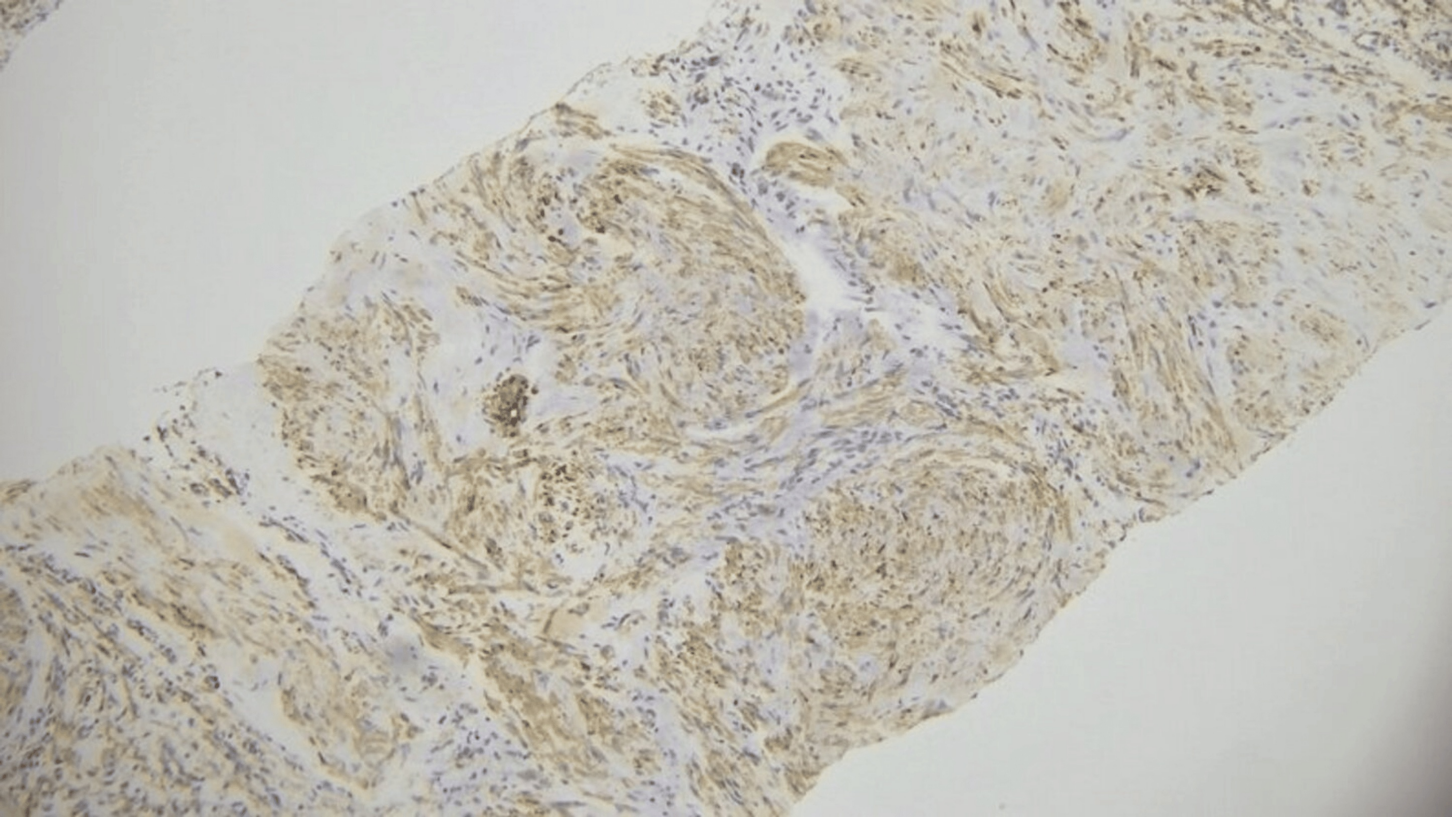
Smooth muscle antigen (SMA) positive (immunohistochemical staining)

**Figure 6:**
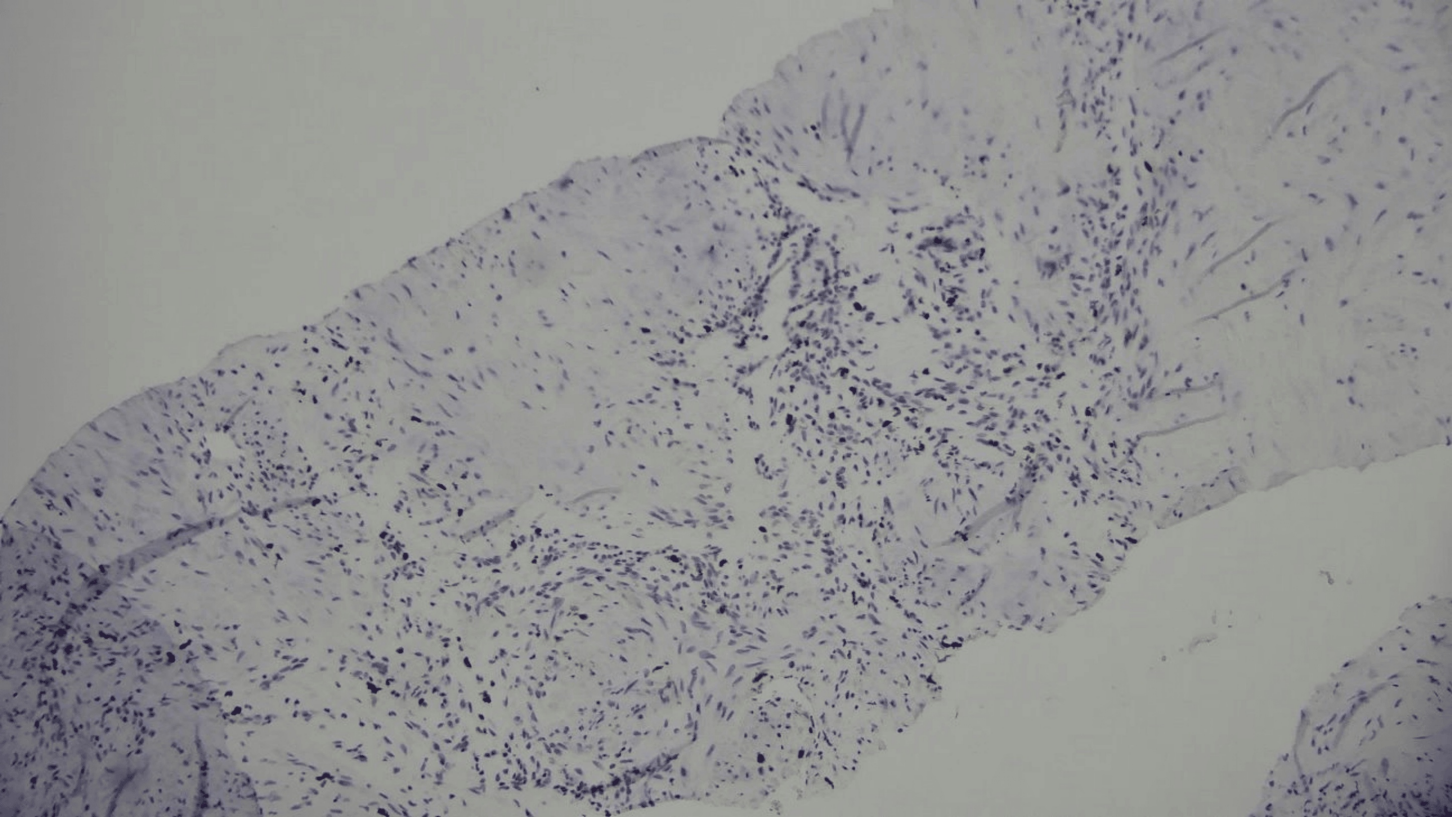
Ki67 low

Subsequently, a US-guided core needle biopsy of the mass was performed for pathologic diagnosis that revealed a bland spindle cell proliferation with interspersed zones of increased collagen. No epithelial component, no cytologic atypia, and no necrosis were identified. Immunohistochemical stains were performed on core biopsy tissue positive for smooth muscle actin and negative for CD34, S100, desmin, p63, and calponin. Ki67 staining was not robust (Figures 3-6).

The overall findings were consistent with myofibroblastic proliferation. Her case was presented in a multidisciplinary tumor board for pathologic and radiologic review and further management plan. Based on the tumor board discussion, it was established that the mass was benign in etiology, and the growth of the mass was slow based on comparative evaluation of previous and current imaging. The consensus recommendation was surgical resection for optimal local control of the mass vs observation with short-interval imaging. 

## Discussion

Therapeutic use of RAI started in 1942 when it was administered for hyperthyroidism treatment [4]. RAI was first used successfully in 1949 for metastatic thyroid cancer at the Royal Cancer Hospital, and patients survived for many years [5]. Since then, the use of RAI for thyroid cancer is well established. The incidence of thyroid cancer has been rising since the 1970s, as has the usage of RAI [1]. The population of long-term survivors of thyroid cancer with previous RAI treatment is also growing. As thyroid cancer is usually diagnosed in children and young patients, there has been concern about the risk of adverse effects of RAI, especially secondary malignancies. This population subset is particularly susceptible to second malignancies because of the longer life span. SEER-based data analysis of patients with well-differentiated thyroid carcinomas who were treated with RAI (between 1973 and 2022) showed that, at least with 20 years of follow-up, the risk of secondary malignancies in patients treated with RAI was higher than in the general population [6].

These secondary malignancies include carcinomas of the salivary gland, stomach, kidneys, breast, lung, and leukemias. However, in our case report, the patient developed a tumor consistent with myofibroblastic proliferation (MFP) in the neck more than 20 years after treatment with RAI. Given the history of RAI exposure, the long interval since exposure and the very close proximity of the MFP directly adjacent to the thyroid all support that the MFP is likely a secondary result of the RAI exposure.

MFP is an abnormal growth of myofibroblasts that are specialized cells with features of both fibroblasts and smooth muscle cells. Abnormal growth of myofibroblasts can be triggered by a variety of insults, including chronic inflammation, infection, or injury, leading to tissue scarring and subsequent proliferation, causing MFP. Treatment generally involves surgical excision, but there is evidence of recurrences after the resection. Steroids and non-steroid anti-inflammatory drugs are also sometimes employed. While most of the MFPs are benign, there is a small risk of malignant transformation and invasion into the local structures, causing problems, especially in recurrent settings and atypical cases [7,8]

To our knowledge, this is the first case report of myofibroblastic proliferation in the neck from radioactive iodine exposure.

## Conclusions

Radioactive iodine is an important part of the management of thyroid cancers, but is not without risks, especially in the younger patient population. This case report highlights the risk of secondary malignancy from RAI exposure in earlier years of life. Given the long latency period and the lesion's anatomical location adjacent to the irradiated thyroid bed, we propose that this benign myofibroblastic proliferation is a previously unacknowledged secondary effect of RAI exposure. While RAI's association with various secondary malignancies is established, this represents the first reported case of MFP in this context, emphasizing the broad potential for delayed, non-malignant proliferative changes following therapeutic radiation and reinforcing the importance of meticulous long-term follow-up for all patients treated with RAI.
